# Draft Genome Sequences of *Methanobrevibacter curvatus* DSM11111, *Methanobrevibacter cuticularis* DSM11139, *Methanobrevibacter filiformis* DSM11501, and *Methanobrevibacter oralis* DSM7256

**DOI:** 10.1128/genomeA.00617-16

**Published:** 2016-06-23

**Authors:** Anja Poehlein, Henning Seedorf

**Affiliations:** a Genomic and Applied Microbiology & Göttingen Genomics Laboratory, Georg-August University, Göttingen, Germany; b Temasek Life Sciences Laboratory, Singapore

## Abstract

Here, the draft genome sequences of four different *Methanobrevibacter* species are presented. Three of the *Methanobrevibacter* species (*M. curvatus, M. cuticularis*, and *M. filiformis*) have been isolated from the termite hindgut, while *M. oralis* was isolated from human subgingival plaque.

## GENOME ANNOUNCEMENT

*Methanobacteriales*, in particular members of the genera *Methanobrevibacter*, have been shown to be some of the most abundant methanogenic archaea in various intestinal environments, including the human gut (1), the termite hindgut (2), and the ovine and bovine rumen (3). Recent studies have indicated that *Methanobrevibacter* species may not only be contributing to greenhouse gas emissions from farm animals (4, 5), but may also have effects on human physiology and health (6, 7). It is therefore of great interest to gain a better understanding of how different *Methanobrevibacter* species have adapted to specific host environments at the molecular level. Genome sequences of *Methanobrevibacter* species have been obtained so far for strains from the human intestinal tract and the rumen (8–12), but not from any insect guts or the human oral cavity. *Methanobrevibacter oralis* DSM7256, isolated from the human subgingival plaque (13), is also the first sequenced representative of all human oral methanogens.

Genomic DNA was ordered by the DSMZ (Braunschweig) or was isolated using the MasterPure complete DNA purification kit (Epicentre, Madison, WI, USA). The extracted DNA was used to generate Illumina-shotgun libraries according to the manufacturer’s protocol (Illumina, San Diego, CA, USA). Sequencing was conducted using a MiSeq and MiSeq reagent kit v3 (2 × 300 bp paired end) as recommended by the manufacturer (Illumina). Sequencing resulted in 1,934,710 *(M. filiformis*), 1,983,778 (*M. oralis*), 3,298,762 *(M. curvatus*), and 3,533,158 paired end reads *(M. cuticularis*), respectively. Trimmomatic 0.32 (14) was used to filter low-quality reads and for clipping of adapter contaminations. The assembly was performed with the SPAdes genome assembler software 3.6.2 (15). Coverages were determined using QualiMap version 2.1 (15, 16) and automatic annotation was performed using the software tool PROKKA (17). General genome features are listed in Table 1.

**TABLE 1:** Genome features and GenBank accession numbers of sequenced strains

Strain	Genome size (bp)	G+C content (%)	No. of scaffolds (>500 bp)	No. of CDSs^*a*^	No. of rRNAs	No. of tRNAs	Accession no.
*M. filiformis* DSM 11501	2,606,143	26.99	295	1,933	3	29	LWMT00000000
*M. oralis* DSM 7256	2,140,433	27.71	136	1,994	5	24	LWMU00000000
*M. curvatus* DSM 11111	2,414,608	25.72	232	1,969	4	31	LWMV00000000
*M. cuticularis* DSM 11139	2,608,702	26.79	169	2,061	3	30	LWMW00000000

a CDSs, coding sequences.

Sequencing the genomes of the four different *Methanobrevibacter* genomes provides reference sequences for comparative analyses with other *Methanobrevibacter* genomes and may reveal adaptive traits of *Methanobrevibacter* species to different environments. Some characteristic features and differences between *Methanobrevibacter* species are already apparent from formal description of the type strains, e.g., presence of catalase activity in the three *Methanobrevibacter* species from the termite hindgut (18, 19). The genome sequences allow the identification of the potential molecular basis of this enzyme activity: A monofunctional heme-depended catalase similar to the enzyme purified from *M. arboriphilus* (20). The gene encoding this enzyme is present in each of the genomes of the three *Methanobrevibacter* species isolated from the termite hindgut, but appears to be absent from the genome of *M. oralis*.

### Nucleotide sequence accession numbers.

These whole-genome shotgun projects have been deposited at DDBJ/EMBL/GenBank under the accession numbers listed in Table 1. The versions described here are the first versions.

## References

[1] Dridi B, Henry M, El Khéchine A, Raoult D, Drancourt M 2009 High prevalence of *Methanobrevibacter smithii* and *Methanosphaera stadtmanae* detected in the human gut using an improved DNA detection protocol. PLoS One 4:e7063. doi:10.1371/journal.pone.0007063. 19759898 PMC2738942

[2] Ohkuma M, Noda S, Kudo T 1999 Phylogenetic relationships of symbiotic methanogens in diverse termites. FEMS Microbiol Lett 171:147–153. doi:10.1111/j.1574-6968.1999.tb13425.x. 10077839

[3] Janssen PH, Kirs M 2008 Structure of the archaeal community of the rumen. Appl Environ Microbiol 74:3619–3625. doi:10.1128/AEM.02812-07. 18424540 PMC2446570

[4] Seedorf H, Kittelmann S, Henderson G, Janssen PH 2014 Rim-DB: a taxonomic framework for community structure analysis of methanogenic archaea from the rumen and other intestinal environments. PeerJ 2:e494. doi:10.7717/peerj.494. 25165621 PMC4137658

[5] Shi W, Moon CD, Leahy SC, Kang D, Froula J, Kittelmann S, Fan C, Deutsch S, Gagic D, Seedorf H, Kelly WJ, Atua R, Sang C, Soni P, Li D, Pinares-Patiño CS, McEwan JC, Janssen PH, Chen F, Visel A, Wang Z, Attwood GT, Rubin EM 2014 Methane yield phenotypes linked to differential gene expression in the sheep rumen microbiome. Genome Res 24:1517–1525. doi:10.1101/gr.168245.113. 24907284 PMC4158751

[6] Lepp PW, Brinig MM, Ouverney CC, Palm K, Armitage GC, Relman DA 2004 Methanogenic *Archaea* and human periodontal disease. Proc Natl Acad Sci USA 101:6176–6181. doi:10.1073/pnas.0308766101. 15067114 PMC395942

[7] Million M, Maraninchi M, Henry M, Armougom F, Richet H, Carrieri P, Valero R, Raccah D, Vialettes B, Raoult D 2012 Obesity-associated gut microbiota is enriched in *Lactobacillus reuteri* and depleted in *Bifidobacterium animalis* and *Methanobrevibacter smithii*. Int J Obes 36:817–825. doi:10.1038/ijo.2011.153. PMC3374072 21829158

[8] Leahy SC, Kelly WJ, Altermann E, Ronimus RS, Yeoman CJ, Pacheco DM, Li D, Kong Z, McTavish S, Sang C, Lambie SC, Janssen PH, Dey D, Attwood GT 2010 The genome sequence of the rumen methanogen *Methanobrevibacter ruminantium* reveals new possibilities for controlling ruminant methane emissions. PLoS One 5:e8926. doi:10.1371/journal.pone.0008926. 20126622 PMC2812497

[9] Hansen EE, Lozupone CA, Rey FE, Wu M, Guruge JL, Narra A, Goodfellow J, Zaneveld JR, McDonald DT, Goodrich JA, Heath AC, Knight R, Gordon JI 2011 Pan-genome of the dominant human gut-associated archaeon, *Methanobrevibacter smithii*, studied in twins. Proc Natl Acad Sci USA 108:4599–4606. doi:10.1073/pnas.1000071108. 21317366 PMC3063581

[10] Leahy SC, Kelly WJ, Li D, Li Y, Altermann E, Lambie SC, Cox F, Attwood GT 2013 The complete genome sequence of *Methanobrevibacter* sp. AbM4. Stand Genomic Sci 8:215–227. doi:10.4056/sigs.3977691. 23991254 PMC3746419

[11] Lee J-H, Rhee M-S, Kumar S, Lee G-H, Chang D-H, Kim D-S, Choi S-H, Lee D-W, Yoon M-H, Kim B-C 2013 Genome sequence of *Methanobrevibacter* sp. strain JH1, isolated from rumen of Korean native cattle. Genome Announc 1(1):e00002-13. doi:10.1128/genomeA.00002-13. 23469331 PMC3587918

[12] Kelly WJ, Li D, Lambie SC, Cox F, Attwood GT, Altermann E, Leahy SC 2016 Draft genome sequence of the rumen methanogen *Methanobrevibacter olleyae* YLM1. Genome Announc 4(2):e00232-00216. doi:10.1128/genomeA.00232-16. 27056228 PMC4824261

[13] Ferrari A, Brusa T, Rutili A, Canzi E, Biavati B 1994 Isolation and characterization of *Methanobrevibacter oralis* sp. nov. Curr Microbiol 29:7–12. doi:10.1007/BF01570184.

[14] Bolger AM, Lohse M, Usadel B 2014 Trimmomatic: a flexible trimmer for Illumina sequence data. Bioinformatics 30:2114–2120. doi:10.1093/bioinformatics/btu170. 24695404 PMC4103590

[15] Bankevich A, Nurk S, Antipov D, Gurevich AA, Dvorkin M, Kulikov AS, Lesin VM, Nikolenko SI, Pham S, Prjibelski AD, Pyshkin AV, Sirotkin AV, Vyahhi N, Tesler G, Alekseyev MA, Pevzner PA 2012 SPAdes: a new genome assembly algorithm and its applications to single-cell sequencing. J Comput Biol 19:455–477. doi:10.1089/cmb.2012.0021. 22506599 PMC3342519

[16] García-Alcalde F, Okonechnikov K, Carbonell J, Cruz LM, Götz S, Tarazona S, Dopazo J, Meyer TF, Conesa A 2012 Qualimap: evaluating next-generation sequencing alignment data. Bioinformatics 28:2678–2679. doi:10.1093/bioinformatics/bts503. 22914218

[17] Seemann T 2014 Prokka: rapid prokaryotic genome annotation. Bioinformatics 30:2068–2069. doi:10.1093/bioinformatics/btu153. 24642063

[18] Leadbetter JR, Breznak JA 1996 Physiological ecology of *Methanobrevibacter cuticularis* sp. nov. and *Methanobrevibacter curvatus* sp. nov., isolated from the hindgut of the termite *Reticulitermes flavipes*. Appl Environ Microbiol 62:3620–3631. 8837417 10.1128/aem.62.10.3620-3631.1996 PMC168169

[19] Leadbetter JR, Crosby LD, Breznak JA 1998 *Methanobrevibacter filiformis* sp. nov., a filamentous methanogen from termite hindguts. Arch Microbiol 169:287–292. doi:10.1007/s002030050574. 9531629

[20] Shima S, Sordel-Klippert M, Brioukhanov A, Netrusov A, Linder D, Thauer RK 2001 Characterization of a heme-dependent catalase from *Methanobrevibacter arboriphilus*. Appl Environ Microbiol 67:3041–3045. doi:10.1128/AEM.67.7.3041-3045.2001. 11425719 PMC92978

